# Reconstruction of Photoreceptor Outer Layers after Steroid Therapy in Solar Retinopathy

**DOI:** 10.1155/2018/7850467

**Published:** 2018-06-11

**Authors:** Masaki Nakamura, Koji Komatsu, Satoshi Katagiri, Takaaki Hayashi, Tadashi Nakano

**Affiliations:** ^1^Department of Ophthalmology, The Jikei University School of Medicine, Tokyo, Japan; ^2^Department of Ophthalmology, Katsushika Medical Center, The Jikei University School of Medicine, Tokyo, Japan

## Abstract

**Purpose:**

To report the clinical course of solar retinopathy after steroid therapy.

**Case Presentation:**

A 45-year-old male gazed at the sun and noticed bilateral central scotoma and decreased vision after the episode. After 7 weeks from onset, ophthalmic examinations were firstly performed. Decimal best corrected visual acuity (BCVA) was decreased to 0.8 and 0.7 in the right and left eyes. Funduscopy showed a tiny, yellowish spot in the fovea bilaterally. Corresponding to the lesion, optical coherence tomography (OCT) images showed an elevated and blurred ellipsoid zone and loss of the interdigitation zone. A posterior sub-Tenon triamcinolone injection in the right eye and oral prednisolone therapy were performed as a medication. BCVA was improved to 1.2 and 1.0 in the right and left eyes at 9 weeks after medication. OCT images showed ellipsoid zone was gradually improved bilaterally, which became nearly normal at 4 weeks in the right eye and at 21 weeks in the left eye. The loss of the interdigitation zone remained at 12 weeks in the right eye and at 21 weeks at the left eye.

**Conclusions:**

We described a case with solar retinopathy who exhibited anatomical recovery of the photoreceptor outer layers by steroid therapy, started after 7 weeks from onset.

## 1. Introduction

Symptoms of solar retinopathy include decreased vision, central scotoma, photophobia, and chromatopsia after gazing at the sun during solar eclipses or a typical sunny day [1]. Histopathological studies have confirmed that energy from sunlight is absorbed into melanosomes of the retinal pigment epithelium, which can lead to solar retinopathy with thermal and photochemical damage to the photoreceptor cells and retinal pigment epithelium [2]. Clinical studies using multimodal imaging including optical coherence tomography (OCT) and fundus autofluorescence imaging (FAF) have reported finding changes of the photoreceptor cells and retinal pigment epithelium during the acute phase and an increased choroidal thickness due to photic injury during the chronic phase [3–5].

We report a case of solar retinopathy in which the subject initially underwent ophthalmic examinations at 7 weeks after the onset of decreased vision and subsequent administration of steroid therapy. The purpose of this study was to report the clinical course of solar retinopathy during the subacute phase after undergoing steroid therapy.

## 2. Case Presentation

**Figure 1:**
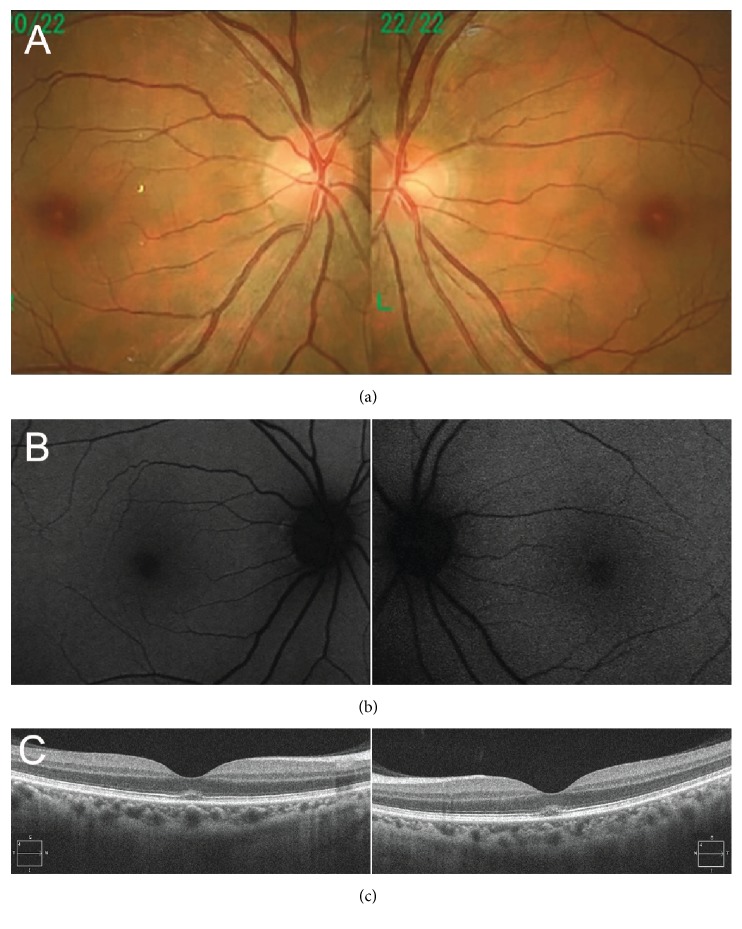
Fundus photographs, fundus autofluorescence imaging (FAF), and optical coherence tomography (OCT) images obtained during the first examination. (a) Fundus photograph showed a tiny, yellowish spot in the bilateral fovea. (b) FAF showed no abnormalities in either of the eyes. (c) OCT showed a diffused and elevated ellipsoid zone and disrupted interdigitation zone in both foveal areas.

**Figure 2:**
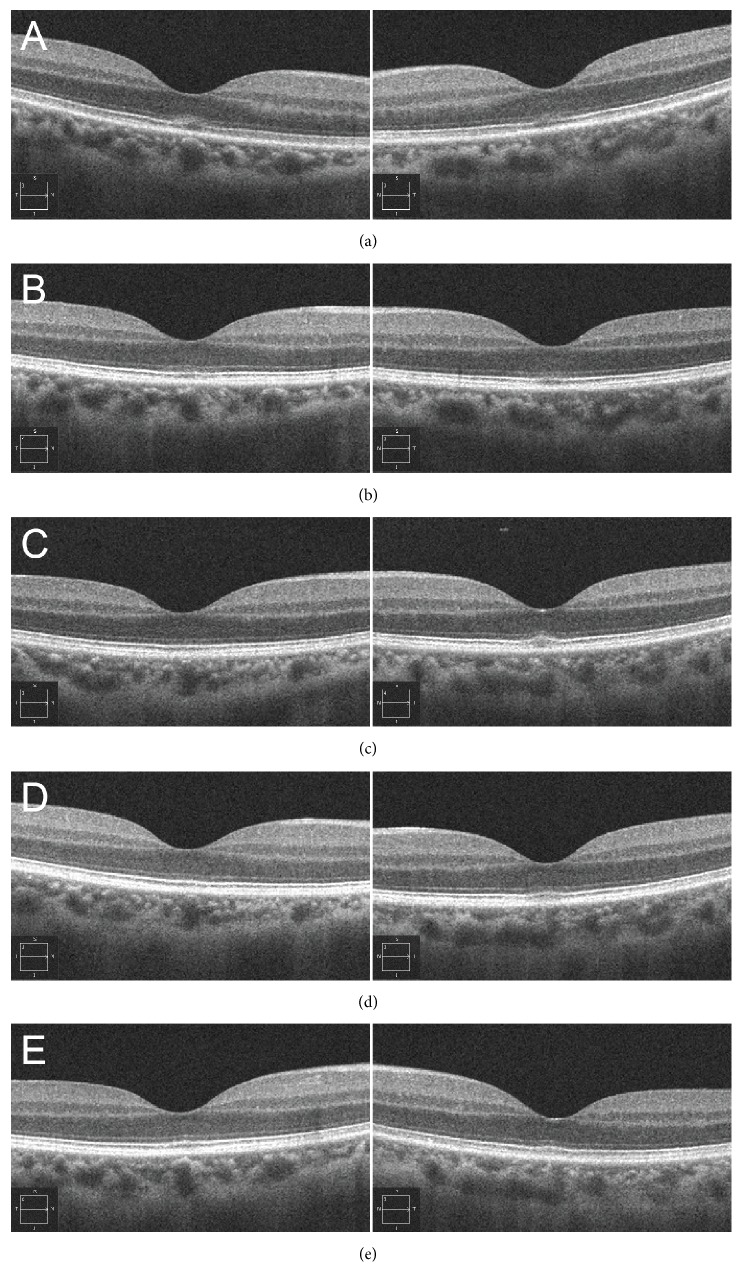
Optical coherence tomography (OCT) images during the follow-up examinations. (a–e) OCT images at 2 (a), 4 (b), 9 (c), 12 (d), and 21 (e) weeks after beginning prednisolone therapy. Ellipsoid zone elevation was improved in both eyes after 2 weeks. Diffused ellipsoid zone gradually improved and became almost normal after 4 weeks in the right eye and after 21 weeks in the left eye. There was observation of the disrupted interdigitation zone after 12 weeks in the right eye and after 21 weeks in the left eye.

A 45-year-old male patient gazed at the sun several times during a baseball game that took place on a sunny day at 7 weeks prior to his first visit to our clinic. Immediately after gazing at the sun, the subject reported having bilateral central scotoma and decreased vision. At the time of the incident, the patient was taking etizolam for a psychiatric condition (panic disorder). At the first visit, his decimal best corrected visual acuity was 0.8 (logMAR conversion: 0.10) (with -3.00 diopters, cylinder -1.00 diopters axis 5°) in the right eye and 0.7 (logMAR conversion: 0.15) (with -3.00 diopters, cylinder -1.00 diopters axis 180°) in the left eye. Slit lamp examinations showed no abnormalities in the anterior segments and media of both eyes. Fundus examinations showed a tiny, yellowish spot in the fovea bilaterally (Figure 1(a)). FAF (Spectralis HRA; Heidelberg Engineering, Heidelberg, Germany) (Figure 1(b)), fluorescein angiography, and indocyanine green angiography all indicated that there were no remarkable abnormalities in either of the eyes. OCT (Cirrus HD-OCT; Carl Zeiss Meditec AG, Dublin, CA, USA) images showed an elevated and blurred ellipsoid zone along with loss of the interdigitation zone at the foveal area bilaterally (Figure 1(c)). There was also no vitreomacular adhesion or traction seen in either of the eyes (Figure 1(c)). When the findings were taken together, the patient was diagnosed with solar retinopathy due to the characteristic symptoms and bilateral findings present after an episode of sun gazing. Treatment was started at the first visit, with the patient given a posterior sub-Tenon triamcinolone injection in his right eye followed by being placed on oral prednisolone therapy (30 mg per day) on the same day. The prednisolone therapy was decreased over a 12-week tapering period. There were no changes noted in the decimal best corrected visual acuity at 2, 4, and 6 weeks after starting the medication. However, at 9 weeks, there was improvement to 1.2 in the right eye and 1.0 in the left eye, with this good visual acuity sustained and observed at the examinations at 12 and 21 weeks. Fundus examinations performed at 12 weeks after the initial treatment showed the tiny, yellowish spots were diminished in both eyes. Sequential OCT images obtained during the follow-up examinations showed that the blurred ellipsoid zone that was visible in both eyes at 2 weeks after initiation of the therapy along with the elevated ellipsoid zone both improved to nearly normal at 4 weeks in the right eye and at 21 weeks in the left eye. However, loss of the interdigitation zone was observed after 12 weeks in the right eye and after 21 weeks in the left eye (Figure 2).

## 3. Discussion

Fundus examinations, fluorescein angiography, OCT, and FAF have all shown that damage to the retina during the acute phase (within a few hours of onset) of solar retinopathy generally exhibits abnormalities of the photoreceptor cells and retinal pigment epithelium at the fovea [3–6]. Over time, these foveal changes become inconspicuous and are followed by recovery of vision [4, 7]. OCT images obtained during the first examination of the present case showed the presence of tiny, yellowish spots at the fovea and ellipsoid zone/interdigitation zone abnormalities. However, there were no abnormalities found in the fluorescein angiography, indocyanine green angiography, or FAF images. Our evaluations found that the damage was limited to the photoreceptor outer layers. A previous histopathological study reported that retinal pigment epithelium cells rapidly regenerate and photoreceptor cells begin to disappear and degenerate sometime after the initial exposure to sunlight [2]. Thus, our present findings are consistent with the presence of the subacute phase (around 1 month from onset) of solar retinopathy. It is possible that the retinal pigment epithelium damage could have been present at the acute phase and then recovered during the 7 weeks prior to the patient's first examination.

Previous studies have reported that most solar retinopathy cases have a complete recovery of vision within a few weeks or months, although there are some cases in which there is decreased vision and/or central scotoma over a long period of time [8, 9]. The differences in the clinical course between these two groups (cases with rapid visual recovery versus cases with prolonged visual damage) are thought to be dependent on the degree of the retinal damage and multiple associated factors, e.g., degree of sun exposure, geographical wavelength, race (differences due to choroidal and retinal pigmentation), and body temperature [1]. Atmaca et al. examined 40 eyes with eclipse retinopathy and decreased best corrected visual acuity under 1.0 within a week of the onset and reported that 14 eyes exhibited visual recovery to 1.0 within 1 month, whereas 26 eyes had not reached 1.0 at 18 months after the onset [9]. Our present case did not exhibit a complete visual recovery to 1.0 at 7 weeks after the onset. Thus, in order to achieve visual recovery in these patients, some type of interventional therapy might be necessary. Based on this supposition, we decided to perform steroid therapy in this patient.

Although an effective treatment for solar retinopathy has yet to be established, the use of steroid therapy has been reported in a few solar retinopathy cases. Bruè et al. evaluated two cases with solar retinopathy who were subsequently treated by systemic steroid administration during the acute phase [10]. After being immediately treated with steroids after the diagnosis, both cases exhibited some visual recovery within 1 week after the sun exposure, with the patients able to achieve a recovery of vision to 20/20 after 4 and 6 months of treatment [10]. Due to this prompt recovery of vision in both cases, the authors hypothesized that inflammation might exist within the retina after photic injury. Our present case also showed gradual recovery of his vision in both eyes after undergoing oral prednisolone therapy, although it was more prominent in the right eye that received an additional posterior sub-Tenon triamcinolone injection. Even though our case was first given steroid medication at 7 weeks after onset, photoreceptor layer improvements were noted in the OCT images at 2 weeks after starting the medication. These findings indicate that inflammation might be occurring in solar retinopathy cases and thus the visual recovery period could be shortened by the administration of steroids. Further studies will need to be undertaken in order to clarify whether or not steroid therapy is effective in cases with prolonged solar retinopathy.

This report describes a patient with solar retinopathy who exhibited substantial visual improvement and anatomical recovery of the photoreceptor outer layers following systemic steroid therapy despite 7 weeks passing from the initial sun gazing damage. The present results suggest that steroid therapy might be an effective treatment for shortening the visual recovery period.
